# Positron emission tomography imaging of serotonin degeneration and beta-amyloid deposition in late-life depression evaluated with multi-modal partial least squares

**DOI:** 10.1038/s41398-021-01539-9

**Published:** 2021-09-13

**Authors:** Gwenn S. Smith, Clifford I. Workman, Hillary Protas, Yi Su, Alena Savonenko, Hiroto Kuwabara, Neda F. Gould, Michael Kraut, Jin Hui Joo, Ayon Nandi, Dimitri Avramopoulos, Eric M. Reiman, Kewei Chen

**Affiliations:** 1 Division of Geriatric Psychiatry and Neuropsychiatry, Department of Psychiatry and Behavioral Sciences, Johns Hopkins University School of Medicine, Baltimore, MD USA; 2 Division of Nuclear Medicine and Molecular Imaging, Russell H. Morgan Department of Radiology and Radiological Sciences, Johns Hopkins University School of Medicine, Baltimore, MD USA; 3 Banner Alzheimer’s Institute, Phoenix, AZ USA; 4 Department of Pathology (Neuropathology), Johns Hopkins University School of Medicine, Baltimore, MD USA; 5 Division of Neuroradiology, Russell H. Morgan Department of Radiology and Radiological Sciences, Johns Hopkins University School of Medicine, Baltimore, MD USA; 6 Department of Genetic Medicine, Johns Hopkins University School of Medicine, Baltimore, MD USA

**Keywords:** Depression, Molecular neuroscience

## Abstract

Depression in late-life is associated with increased risk of cognitive decline and development of all-cause dementia. The neurobiology of late-life depression (LLD) may involve both neurochemical and neurodegenerative mechanisms that are common to depression and dementia. Transgenic amyloid mouse models show evidence of early degeneration of monoamine systems. Informed by these preclinical data, the hypotheses were tested that a spatial covariance pattern of higher beta-amyloid (Aβ) and lower serotonin transporter availability (5-HTT) in frontal, temporal, and parietal cortical regions would distinguish LLD patients from healthy controls and the expression of this pattern would be associated with greater depressive symptoms. Twenty un-medicated LLD patients who met DSM-V criteria for major depression and 20 healthy controls underwent PET imaging with radiotracers for Aβ ([^11^C]-PiB) and 5-HTT ([^11^C]-DASB). A voxel-based multi-modal partial least squares (mmPLS) algorithm was applied to the parametric PET images to determine the spatial covariance pattern between the two radiotracers. A spatial covariance pattern was identified, including higher Aβ in temporal, parietal and occipital cortices associated with lower 5-HTT in putamen, thalamus, amygdala, hippocampus and raphe nuclei (dorsal, medial and pontine), which distinguished LLD patients from controls. Greater expression of this pattern, reflected in summary 5-HTT/Aβ mmPLS subject scores, was associated with higher levels of depressive symptoms. The mmPLS method is a powerful approach to evaluate the synaptic changes associated with AD pathology. This spatial covariance pattern should be evaluated further to determine whether it represents a biological marker of antidepressant treatment response and/or cognitive decline in LLD patients.

## Introduction

Major depressive disorder in late-life is a risk factor for the development of all-cause dementia [1, 2]. Further, depressive symptoms are a risk factor for cognitive decline in cognitively normal individuals, as well as in individuals with mild cognitive impairment [3, 4]. The treatment of late-life depression (LLD) may be a target for dementia prevention [5, 6]. However, antidepressant treatments, developed for the treatment of younger patients, are effective in about half of patients in late-life [7]. The pathophysiology of LLD is multi-factorial and includes neurochemical, neurodegenerative and cerebrovascular disease mechanisms [8]. A clearer understanding of the neurobiology of LLD may facilitate the identification of individuals at risk for cognitive decline, as well as contribute to the development of more effective symptomatic treatments and strategies for prevention.

Molecular imaging studies of LLD have focused on Alzheimer’s disease (AD) pathology, employing positron emission tomography (PET) radiotracer imaging of beta-amyloid (Aβ), as well as fluid biomarkers [9–12]. PET studies have shown either no difference, higher or lower Aβ in LLD patients compared to healthy controls. When greater Aβ was observed in LLD patients than controls (in the absence of a mild cognitive impairment (MCI) or AD diagnosis), Aβ was detected in a more localized neuroanatomic distribution compared to MCI or AD [10, 13]. Studies in cognitively normal individuals have shown an association between depressive symptoms and greater Aβ and Tau [14, 15]. Aβ measured with PET and plasma CSF tau/Aβ42 ratios was associated with the development of depressive symptoms in longitudinal studies of healthy controls [16, 17]. While there are discrepancies in the observation of greater Aβ in LLD patients, evidence suggests that in cognitively normal individuals, Aβ and depressive symptoms interact synergistically and are associated with cognitive decline [18].

While PET measures of Aβ and Tau show independent associations with cognitive deficits and cognitive decline in healthy controls and individuals with MCI, their predictive value is increased by including measures associated with neuronal dysfunction or degeneration (e.g., measures of cerebral glucose metabolism or gray matter volume) [19–22]. Studies comparing LLD patients to controls have demonstrated cortical and hippocampal atrophy and increased glucose metabolism in cortical regions that are hypometabolic both in AD patients and in individuals at genetic risk for AD [23–25]. To understand the neurobiology of LLD, multi-radiotracer imaging may provide information regarding AD pathology as well as detecting associated molecular mechanisms that have been implicated in both depression and neurodegenerative disease.

In considering molecular mechanisms associated with AD pathology, degeneration of monoamine systems (especially the serotonin system) has been observed in normal aging, MCI and AD, as well as depression [26, 27]. In AD, serotonergic deficits are greater and more widespread than deficits in other monoaminergic and cholinergic systems [28, 29]. Molecular imaging studies of the serotonin system have focused on measuring serotonin transporter availability (5-HTT). 5-HTT is a more specific marker of serotonin degeneration than serotonin receptors (e.g., 5-HT1a or 5-HT2a) as receptors are localized also on the terminals of non-serotonergic neurons [30]. Lower 5-HTT in depressed patients across the lifespan and in neurodegenerative diseases has been shown [31–33]. Modest decreases in 5-HTT have been observed in temporal cortical and limbic regions (amygdala, hippocampus) in LLD patients relative to controls, in contrast to the lack of a difference in serotonin receptor availability (5-HT1a and 5-HT2a) [32, 34, 35]. Lower 5-HTT has been observed also in patients with MCI and AD [33, 36].

The relationship between serotonin degeneration and AD pathology has been appreciated in preclinical studies. In a transgenic model of β-amyloidosis (APPswe/PS1dE9 mice), reduced serotonergic and noradrenergic fiber densities in the cortex and hippocampus were observed that occurred in parallel to the onset of deficits in episodic memory (12 months) and worsened at the onset of reference memory deficits and anxiety (18 months) [37]. Substantial cortical Aβ occurred later (18–24 months) at a time when cell loss in the subcortical monoaminergic nuclei (raphe nuclei, locus coeruleus) was observed. This pattern of monoamine degeneration was similar to that observed in AD [26]. Further analyses in this and similar transgenic amyloid mouse models showed loss of 5-HTT and 5-HT1b receptors, decreased serotonin release and an increase in the Aβ-related inflammatory response that was associated with change in these serotonergic markers [38, 39].

In the present study, the association between Aβ and 5-HTT in LLD patients and healthy controls was studied with PET imaging using well-characterized radiotracers for Aβ (N-methyl-[11 C]2-(4’-methylaminophenyl)-6-hydroxybenzothiazole, [^11^C]-PiB) and for 5-HTT ([^11^C]-3-amino-4-(2-dimethylaminomethyl-phenylsulfanyl)-enzonitrile, [^11^C]-DASB) [40, 41]. The voxel-wise, multi-modal partial least square (mmPLS) analytic technique was applied to identify the spatial covariance pattern of Aβ and 5-HTT that maximally distinguished LLD patients from controls [42]. The hypotheses were tested that a spatial covariance pattern of higher Aβ and lower 5-HTT in frontal, temporal and parietal cortical regions would differentiate LLD patients from healthy controls. Greater expression of this pattern was predicted to correlate with greater depressive symptoms.

## Materials and methods

### Participant screening and selection

LLD patients were recruited from advertisements in the community. Controls were recruited from advertisements or from the Johns Hopkins University Alzheimer’s Disease Research Center (2P50AG005146). To determine study eligibility, potential LLD patients and controls underwent screening that included physical and neurological examination, laboratory testing and toxicology screening and psychiatric and neuropsychological evaluations. A Structured Clinical Interview for DSM-V was administered by a clinical psychologist (NG) [43], along with the antidepressant treatment history form [44], Clinical Dementia Rating scale (CDR) and a Mini Mental State Examination (MMSE) [45, 46]. LLD patients were enrolled if they were over age 60, had a DSM-V diagnosis of current major depressive episode (non-bipolar, non-psychotic) and a score of 17 or higher on the Hamilton Depression Rating Scale (HDRS) [47]. Participants were excluded from enrollment based on the following criteria: (1) a history of active neurological or Axis I psychiatric disorders (including dementia), except for a diagnosis of current major depressive episode (non-bipolar, non-psychotic) in the LLD patients; (2) not medically stable (i.e., poorly controlled medical conditions including hypertension and/or diabetes); (3) a positive toxicology screening and/or use of psychotropic drugs or medications with central nervous system effects (e.g., antihistamines, cold medications) within 2 weeks prior to enrollment and (4) contraindications for undergoing magnetic resonance imaging (MRI) scans (e.g., pacemaker, metal implants, aneurism clamps). The study protocol and consent forms were approved by the Institutional Review Board and the Radiation Research Committee of the Johns Hopkins University School of Medicine. Participants received both a transcribed and verbal description of the study and written informed consent was obtained.

### Genotyping

Apolipoprotein E (APOE) genotyping was performed (in the laboratory of DA) using polymerase chain reaction amplification of genomic DNA digestion with Hhal restriction enzyme and gel electrophoresis, as described previously [48].

### MR imaging procedures

MRI scans of the brain were acquired within a week before the PET scans at the F. M. Kirby Research Center for Functional Brain Imaging of the Kennedy Krieger Institute, as described previously [13, 32]. A Phillips 3.0 T Achieva MRI instrument was used with an 8-channel head coil (Philips Medical Systems, Best, Netherlands). The magnetization-prepared rapid acquisition with gradient-echo (MPRAGE) pulse sequence (TE = 4, TR = 8.9, flip angle = 8 degrees, NSA = 1, 0.7 mm isotropic voxel size) was used for PET image processing.

### PET imaging acquisition, quantification and preprocessing

PET scans were performed at the PET Center of the Russell H. Morgan Department of Radiology, Johns Hopkins University School of Medicine. The scanner used was a second-generation High Resolution Research Tomograph scanner (HRRT, Siemens Healthcare, Knoxville, TN), a cerium-doped lutetium oxyorthosilicate (Lu25i05 [Ce] or LSO) detector-based, dedicated brain PET scanner [49, 50]. The radiotracers [^11^C]-PiB and [^11^C]-DASB, used to measure Aβ and 5-HTT, respectively, were synthesized according to published methods [40, 41]. The PET scans were performed within 1 week of each other. Procedures for PET scan acquisition and reconstruction have been described previously [13, 32].

### PET tracer kinetic modeling, image processing and statistical parametric mapping (SPM) analysis

Regional distribution volume ratio (DVR) values of [^11^C]-PiB were obtained by the multilinear reference tissue method with 2 parameters (MRTM2) [40, 51]. Regional DVR values of [^11^C]-DASB were obtained by the reference tissue graphical analysis method (RTGA) [32, 52, 53]. For both radiotracers, the cerebellar gray matter (excluding the vermis) was used as the reference region The pre-processing of the parametric [^11^C]-PiB and [^11^C]-DASB DVR images was performed with SPM12 (SPM12; Institute of Neurology, London) as described previously [13, 32].

### Multi-modal partial least squares (mmPLS)

In 2009, Chen and colleagues introduced the voxel-based, multi-modal application of the partial least square algorithm [42, 54]. The technique was applied to combine PET studies of cerebral glucose metabolism with structural MRI data to measure ApoE4 gene-dose effects in cognitively normal individuals [55]. These and other studies demonstrated the power of integrating information from different imaging modalities to increase statistical power and to address the issue of multiple comparisons associated with univariate, voxel-wise analyses. There are two types of mmPLS: agnostic and informed mmPLS. In informed mmPLS, the variable of interest (e.g., diagnostic group membership) was directly incorporated into the mmPLS process. Agnostic mmPLS, on the other hand, is performed blind to the variable of interest. Corresponding to each of the two approaches, statistical test procedures were established to objectively assess type-I error related to the variable of interest (e.g., group differences). The algorithm generates images of the co-varying pattern for each modality and a single mmPLS score (subject score) for each person to reflect the strength of this co-varying pattern.

Agnostic mmPLS was used in this study, given that the primary interest was to assess between-group differences and relationship between the neuroimaging-based measures (mmPLS) and depressive symptom ratings. In running the agnostic mmPLS for dual-modality Aβ and 5-HTT PET data, the covariance patterns were estimated blindly over all subjects (no diagnostic group information was used). The between-group difference was tested using the subject scores associated with each of the spatial covariance patterns. First, agnostic mmPLS was run to generate the co-varying patterns between Aβ and 5-HTT. Then, between-group differences and correlations with depressive symptom ratings were evaluated using the subject scores from the mmPLS procedure that reflects the extent to which each subject expressed the spatial covariance pattern.

### Statistical analysis

Between-group differences in the demographic and clinical measures were tested using analyses of variance (ANOVA) for interval variables, and Chi-square test for categorical variables. The general linear model in SPM12 examined voxel-wise group difference for each of the two PET radiotracers separately. To address the issue of multiple comparison correction, the Monte-Carlo simulation procedure developed by Chen and colleagues was applied to test the hypothesis that the number of between-group, voxel-wise differences in Aβ and 5-HTT in the predicted direction (greater Aβ and less 5-HTT in LLD patients than controls) was significantly greater than differences in the opposite direction. This is a method of estimating the overall significance of the entire voxel-wise image that is not influenced by the type I error associated with multiple regional comparisons. This method has been described previously and is similar to methods developed for voxel-based analyses of other neuroimaging modalities (e.g., structural magnetic resonance and Tau imaging) [56, 57]. Mean cortical Aβ values are reported as calculated previously [13].

**Table 1:** Demographic and clinical characteristics of the late-life depressed (LLD) patients and controls.

	Healthy controls (*n* = 20)	LLD Patients (*n* = 20)
Age (years)	65 ± 7	67 ± 6
Sex (F/M)	10/10	10/10
Education (years)	15.5 ± 4	16.5 ± 2
MMSE	29 ± 1	29 ± 1
HDRS	1 ± 1	18 ± 2^a^
BDI	2 ± 3	24 ± 8^b^

*MMSE* modified Mini-Mental State Examination, *BDI* Beck Depression Inventory, *HDRS* Hamilton Depression Rating Scale.

^a^Significant between group difference (*F*(1,39) = 879.3, *P* < 0.001).

^b^Significant between group difference (*F*(1,39) = 149.4, *P* < 0.001).

The mmPLS spatial covariance pattern (as a 3D brain map) was scaled by the brain-map wide standard deviation within the search area to form the z-score map and was displayed with a threshold of *P* = 0.05, as the correction for multiple comparisons is not relevant for the covariance spatial pattern display. Subject scores, which are free of the need for multiple comparisons corrections associated with voxel-wise, mmPLS analyses were generated blinded to group membership, were used to examine the group differences and to identify the related spatial covariance patterns. Realizing that the subject scores are paired, one for each PET radiotracer, group differences were initially examined using the multivariate Hotelling T square test. Realizing also, however, the close correlation between Aβ and 5-HTT for each subject score pair, principal component analysis (PCA) was applied to project the paired subject scores along the direction of the PCA major axis to form a single subject score to which the conventional univariate two-sample independent *t* test could be applied. The integrated univariate subject scores were also used to examine relationships with depression measures. Finally, if more than one spatial covariance pattern was associated with group differences, the formation of a single pattern was attempted by taking a weighted sum with the weights estimated by the general linear model, which included the subject scores of the corresponding patterns as the predictors to distinguish group membership. Brain regions were identified using the AAL and AAL3 atlases to localize significant results in the brainstem [58–60].

## Results

Twenty LLD patients and 20 healthy controls were enrolled in the study. The demographic characteristics, depression, cognitive and clinical measures are shown in Table 1. The groups did not differ significantly in age and sex distribution, years of education or cognitive functioning (MMSE or CDR sum of boxes). One of the controls and no LLD patients were left-handed. All controls received a CDR score of 0 (normal). All but one LLD patient received a CDR score of 0. This patient received a score of 0.5 (MCI: sum of boxes = 1.5 for memory, orientation, judgment and problem-solving subscales). Another patient received a CDR of 0 and a sum of boxes = 0.5 (judgment and problem-solving subscale). In addition, groups were comparable in the number of ApoE4 alleles (1 subject was a homozygote and 3 subjects were heterozygotes in each group). As expected, LLD patients showed significantly higher HDRS and BDI scores than controls (*F*(1,39) = 879.3, *P* < 0.001); *F*(1,39) = 149.4, *P* < 0.001, respectively, within a range of moderate to moderate-to-severe depressive symptoms, respectively. All patients had one prior depressive episode not including the present episode (5 patients with onset before age 65 and 15 patients with onset after age 65). The age at onset of depression was 58 ± 11 years (range 33–75 years) and the duration of the present episode was 14 ± 10 months (range 2–36 months). None of the LLD patients were taking psychotropic drugs at the time of scanning, including antidepressants, antipsychotics and mood stabilizers. Three LLD patients were treated previously with an adequate trial of selective serotonin reuptake inhibitors (SSRIs) in late life, although not within 2 years of enrollment in the study. The number of participants who were taking medications to treat the following medical co-morbidities were: hypertension (7 control/6 patients), cholesterol lowering medications (6 control/6 patients) and oral anti-diabetogenic agents (0 control/2 patients), but none were taking insulin. No patients had pulmonary disease requiring treatment.

### Unimodal, univariate SPM results

**Fig. 1:**
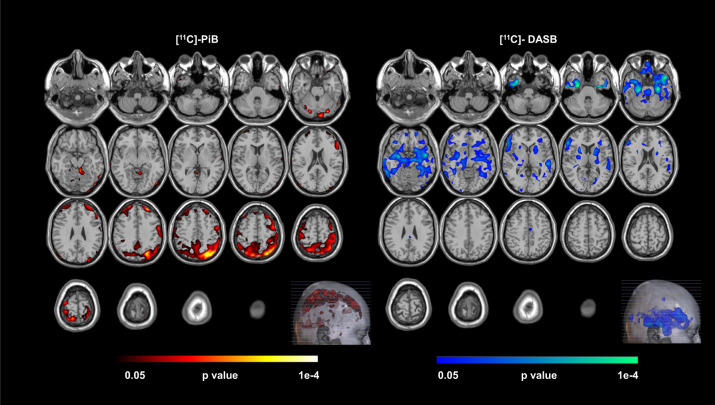
Voxel-wise, statistical parametric mapping (version 12) unimodal analysis for [^11^C]-PiB and [^11^C]-DASB, separately. Higher beta-amyloid (left, hot colored areas) and lower serotonin transporter availability (right, cool colored areas) was observed in Late-Life Depressed (LLD) Patients than in normal controls, applying the Monte-Carlo Simulation Method (uncorrected *P* = 0.005).

SPM12 based voxel-wise analyses were performed separately for Aβ and 5-HTT to examine the group differences between normal controls and LLD patients (Fig. 1, Table 2). For each unimodal SPM voxel-wise analysis, none of the regions survived multiple comparison correction using the commonly reported height threshold *p* = 0.001 and cluster-level threshold of FDR at *P* > 0.05. When examining the omnibus significance using Monte-Carlo simulation, there were 21842 voxels where 5-HTT availability was lower in LLD patients compared to controls, while there were only 3877 voxels in the opposite direction. Using the Monte-Carlo simulation over 1000 runs, the omnibus significance for 5-HTT was *P* < 0.002. Similarly, the omnibus significance was *P* < 0.001 for Aβ for which 22591 and 4582 voxels were observed where Aβ DVR was higher and lower, respectively in LLD patients compared to controls. At a peak-voxel uncorrected threshold of *P* = 0.005, 5-HTT was lower in LLD patients compared to controls superior temporal pole (left), inferior temporal gyrus (right), parahippocampal gyrus (right), fusiform (bilateral), amygdala (bilateral), dorsal, medial and pontine raphe nuclei. Aβ was higher in LLD patients compared to controls in middle frontal gyrus (left), superior and inferior parietal gyrus (left), precuneus (left), angular gyrus (left) and superior occipital gyrus (left). The mean Aβ DVRs for the total cortex and the left precuneus were 1.13 ± 0.63 and 1.24 ± 0.09 for the controls and 1.17 ± 0.16 and 1.31 ± 0.24 for the LLD patients based on volume of interest analysis [13].

**Table 2:** Peak voxel locations identified by statistical parametric mapping (version 12) unimodal analysis.

Brain region	Hemisphere	Atlas coordinates millimeters (MNI) X Y Z	*P* value
Greater beta-amyloid burden in LLD patients compared to controls			
Middle Frontal Gyrus	Left	−28 50 36	9.0E–04
Superior Parietal Gyrus	Left	−26 −74 56	5.4E–04
Precuneus	Left	−4 −80 56	1.3E–03
Inferior Parietal Gyrus	Left	−26 −72 44	3.3E–04
Angular Gyrus	Left	−40 −72 48	4.9E–04
Superior Occipital Gyrus	Left	−26 −72 40	4.0E–04
Lower 5-HTT in LLD patients compared to controls			
Parahippocampal Gyrus	Right	28 −28 −32	2.1E–05
Superior Temporal Pole	Left	−48 14 −24	7.6E–05
Inferior Temporal Gyrus	Right	32 2 −44	1.8E–04
Amygdala	Right	30 −6 −14	4.02E–04
Amygdala	Left	−26 2 −26	1.9E–03
Fusiform Gyrus	Right	30 −12 −36	1.3E–05
Fusiform Gyrus	Left	−28 −12 −32	1.8E–04
Raphe Pontine		−6 −18 −20	6.3E–03
Raphe Median		0 −30 −24	1.3E–02
Raphe Dorsal		4 −28 −12	3.4E–02

Higher Beta-Amyloid (top section) and lower serotonin transporter availability (bottom section) was observed in Late-Life Depressed (LLD) Patients than normal controls, applying the Monte-Carlo Simulation Method (uncorrected *P* = 0.005).

### Dual-modal agnostic mmPLS results

**Fig. 2:**
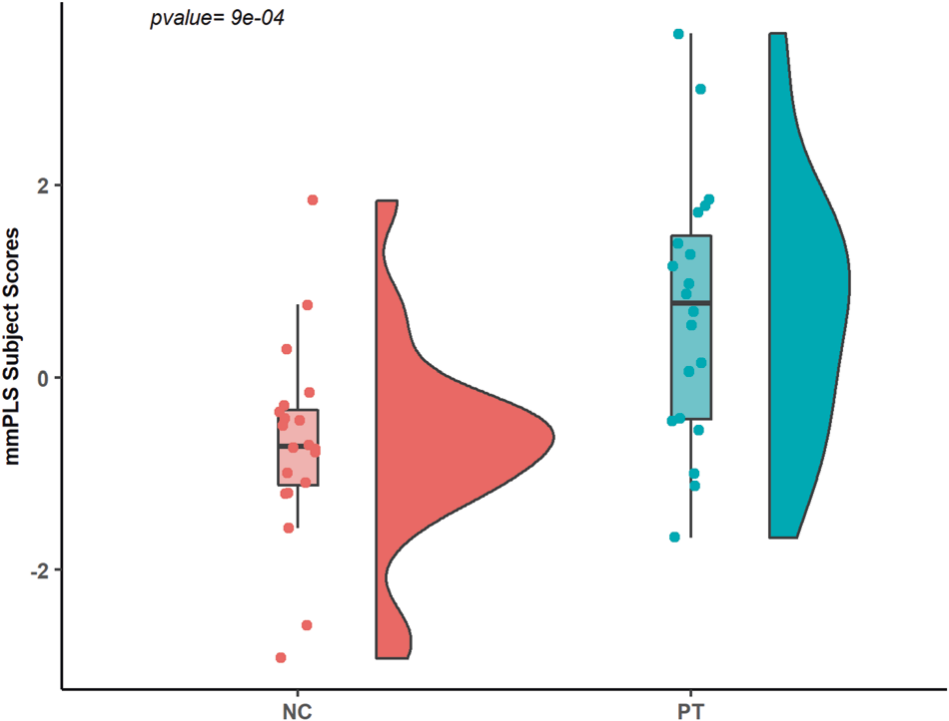
Subject scores for normal controls (NC) and late-life depressed (PT) patients. Subject scores determined by multi-modal partial least squares.

**Fig. 3:**
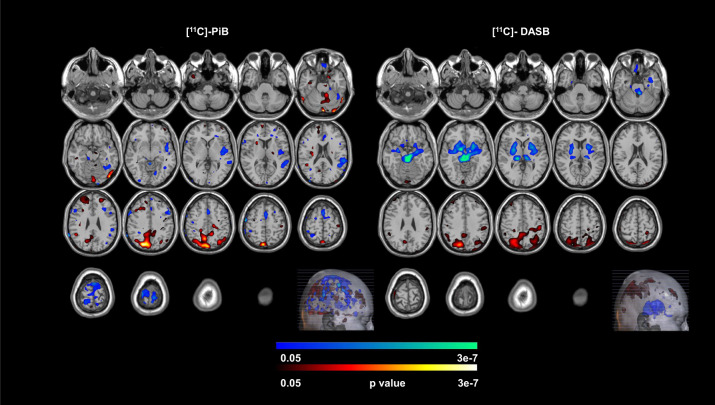
Co-varying spatial patterns between beta-amyloid deposition and serotonin transporter availability. Covarying Spatial patterns determined by Multi-Modal Partial Least Squares in normal controls and late-lifedepressed patients.

Two patterns were identified that showed significant group differences based on the integrated subject score (the main PCA component for the Aβ subject score and 5-HTT subject score, *t* (2,37) = −2.637; *P* = 0.012; *t* (2,37) = −2.110; *P* = 0.046, respectively). To further reduce the possibility of type-I error, the two patterns were combined via the general linear model with the corresponding subject scores as the independent predictors for group differences. The distribution of subject scores for the two groups is shown in Fig. 2. The subject scores are significantly lower in the LLD group than the normal control group (*t* (2,37) = −3.623; *P* = 0.0009) when subject scores for Aβ and 5-HTT were further integrated using PCA. The combined spatial covariance pattern for the two groups and the peak voxel locations for the between-group differences are shown in Fig. 3 and Table 3, respectively. Note that this spatial covariance pattern display is not for statistical inference and there is no need for the correction of multiple comparisons. The combined component demonstrated an association between decreased 5-HTT in the putamen (bilateral), thalamus (bilateral), hippocampus (left), amygdala (bilateral), dorsal, medial and pontine raphe nuclei and higher Aβ in the inferior temporal gyrus (left), precuneus (right), inferior parietal gyrus (left), superior occipital gyrus (right) and cuneus (right).

**Table 3:** The peak voxel locations of the co-varying spatial patterns of Greater Beta-Amyloid and the Serotonin Transporter (5-HTT) determined by multi-modal Partial Least Squares (mmPLS) for the Normal Controls and Late-Life Depressed Patient Groups.

Brain region	Hemisphere	Atlas coordinates millimeters(MNI) X Y Z	*P* value
Greater beta-amyloid in LLD patients compared to controls			
Inferior Temporal Gyrus	Left	−58 −54 −24	2.0E–05
Precuneus	Right	6 −78 52	7.1E–06
Precuneus	Right	6 −78 56	2.2E–05
Inferior Parietal Gyrus	Left	−32 −68 44	8.6E–04
Superior Occipital Gyrus	Right	22 −78 40	1.5E–07
Cuneus	Right	14 −76 36	7.8E–06
Lower 5-HTT in LLD patients compared to controls			
Putamen	Right	26 −4 −4	5.6E–05
Putamen	Left	−22 12 −4	3.7E–05
Thalamus	Right	14 −28 0	9.0E–09
Thalamus	Left	−12 −28 4	1.4E–05
Amygdala	Right	28 −6 −14	2.4E–04
Amygdala	Left	−26 −8 −12	7.7E–05
Hippocampus	Left	−26 −8 −16	3.1E–04
Pontine Raphe		−2 −26 −20	3.1E–07
Dorsal Raphe		0 −28 −14	8.2E–18
Median Raphe		0 −30 −20	1.6E–09

### Correlation results

In addition to the blinded group membership information, the mmPLS pipeline was also agnostic to the depression severity measures. Using the same mmPLS integrated and modality-specific subject scores, the relationship between mmPLS spatial covariance pattern and the depression measures were examined. For all subjects combined, greater expression of the 5-HTT and Aβ covariance pattern was correlated with greater depressive symptoms (Hamilton Depression Rating Scale Score) (HDRS: *r*_s_ = 0.43; *P* = 0.006) and Beck Depression Inventory (BDI *r*_s_ = 0.44; *P* = 0.005; Table 4). The correlation of depressive symptoms with Aβ and 5-HTT were similar (Aβ: *r*_s_ = 0.50; *P* = 0.001 and *r*_s_ = 0.45; *P* = 0.003 and 5-HTT: *r*_s_ = 0.35; *P* = 0.03 and *r*_s_ = 0.42; *P* = 0.007) with HDRS and BDI, respectively. As the mmPLS method is designed to identify the maximal covariance of two spatial patterns, the Aβ and 5-HTT patterns are not entirely independent and the correlations are expected to be similar.

**Table 4:** Correlation between multi-modal partial least squares (mmPLS) determined subject scores and depression severity.

	Integrated		Beta-amyloid		Serotonin transporter availability	
Depression Measure	R_s_	*P* value	R_s_	*P value*	R_s_	*P* value
Hamilton Depression Rating Scale	0.43	0.006	0.50	0.001	0.35	0.03
Beck Depression Inventory	0.44	0.005	0.45	0.003	0.42	0.007

## Discussion

A spatial covariance pattern of lower 5-HTT and higher Aβ was identified that distinguished LLD patients from controls. Specifically, lower 5-HTT in striatal, thalamic (anterior and medial-dorsal nuclei), limbic (amygdala and hippocampus) and raphe nuclei (dorsal, medial, and pontine) were associated with greater Aβ in temporal, parietal and occipital regions. Greater expression of the combined pattern, as well as both Aβ and 5-HTT patterns, was correlated with greater depressive symptoms. Most prior studies have not observed a correlation between depressive symptoms and 5-HTT availability [31]. The correlation between depressive symptoms and 5-HTT pattern may have been significant in the present study because the 5-HTT pattern is not entirely independent of Aβ using this analysis method. The cortical distribution of Aβ in the LLD patients is less in magnitude and extent as compared to the distribution of Aβ in patients with MCI or AD and is similar to other studies in which higher Aβ was observed in LLD patients relative to controls [10, 61, 62]. Importantly, significant associations between the two radiotracers were observed that discriminate between control and LLD groups, while robust between group differences in Aβ and 5-HTT were only observed when examining the omnibus global significance for analyzing each radiotracers separately. Based on the omnibus global assessment of significance, overall higher Aβ and lower 5-HTT density was detected in LLD patients as compared to healthy controls, while the conventional voxel-based comparisons (SPM12), using a stringent statistical threshold, did not detect localized between-group differences. Previous exploratory voxel-wise analyses showed similar results for Aβ and 5-HTT [13, 32]. Although the unimodal approach could not evaluate associations between neurobiological processes, mmPLS, revealed associations between the degree of regional Aβ and 5-HTT.

Several aspects of the spatial covariance pattern are noteworthy. At this relatively early stage of Aβ, decreases in 5-HTT are observed. The raphe nuclei and subcortical and limbic regions are among regions with the highest concentration of 5-HTT in the brain [63, 64]. The raphe nuclei include the cell bodies that synthesize serotonin and are the origin of serotonin innervation throughout the brain [63]. The critical role of serotonin is underscored by the dense serotonin innervation throughout the CNS and the observation in the rat brain that every cell in the cortex is in close proximity to a serotonin containing neuron [63]. The spatial covariance pattern did not include cortical 5-HTT, even though neurodegeneration of the raphe nuclei (suggested by the loss of 5-HTT) would be expected to have a widespread influence on cortical serotonin. 5-HTT may not be sensitive to detecting cortical serotonin dysfunction due to its relatively lower concentration in cortex than the other regions included in the pattern. In contrast, a study in Parkinson’s Disease (PD) patients showed a correlation between higher cortical Aβ and lower cortical 5-HTT and between higher cortical Aβ and lower midbrain 5-HTT. However, the levels of cortical Aβ in the PD patients in this study were comparable to the LLD patients in the present study [65]. A further understanding of the longitudinal regional-specific associations may be critical in understanding mechanisms of LLD and antidepressant response, as well as cognitive decline.

While the present results do not imply causality and may represent two-co-occurring pathologies, interventions targeting either Aβ or 5-HTT have an effect on the other pathology. Aβ immunotherapies show a protective effect on the serotonin system, while interventions for multiple serotonergic targets show a reduction in AD pathology. Aβ immunization attenuated both Aβ and serotonin degeneration in transgenic mice (APPswe/PS1dE9) [66]. Early SSRI treatment prevented the development of AD pathology (Aβ and/or Tau), spatial memory deficits and depression-like symptoms in amyloid (APPswe/PS1dE9) and triple-transgenic mouse models (3xTg-AD) [67, 68]. In the APP/presenilin 1 mouse model and in healthy human subjects, the antidepressant Escitalopram reduced interstitial fluid Aβ and CSF Aβ42 level, respectively [69, 70]. Serotonergic receptor modulators (such as 5-HT4 agonists, 5-HT6 antagonists) that have antidepressant effects reduced Aβ [71, 72]. Importantly, preclinical studies show that serotonergic agents have multiple mechanisms relevant to prevention and symptomatic treatment of both depression and cognitive deficits, in addition to blocking Aβ and Tau, neuroprotection and synaptic plasticity [73]. Thus, an understanding of the role of the serotonin system in relation to AD pathology in LLD may have implications for symptomatic treatment and prevention.

In conclusion, the present study focused on evaluating Aβ relative to a molecular mechanism that is associated with depressive disorders and cognitive deficits and has been linked to Aβ in preclinical studies, serotonin degeneration as reflected by loss of 5-HTT [32, 33, 37]. Using mmPLS, unique associations between Aβ and 5-HTT were demonstrated in LLD patients relative to controls. The results suggest that mmPLS can be applied to understand the synaptic changes potentially associated with Aβ, especially at a relatively early stage of Aβ. The evaluation of 5-HTT in relation to Aβ in a larger, more heterogeneous LLD patient sample would determine whether the spatial covariance pattern may be a sensitive biomarker to identify LLD patients who may respond poorly to SSRI treatment or who may be “at risk” for cognitive decline.
